# Comparison of add-on medications for persistent storage symptoms after α-blocker treatment in BPH patients – a network meta-analysis

**DOI:** 10.1186/s12894-023-01327-1

**Published:** 2023-10-03

**Authors:** Yi-Ting Su, Hsiao-Ling Chen, Jeremy Yuen-Chun Teoh, Vinson Wai-Shun Chan, Wen-Jeng Wu, Hsiang-Ying Lee

**Affiliations:** 1 Department of Urology, Mackay Memorial Hospital, Taipei, Taiwan; 2 Institute of Health and Welfare Policy, National Yang Ming Chiao Tung University, Taipei, Taiwan; 3 Department of Surgery, S.H. Ho Urology Centre, The Chinese University of Hong Kong, Hong Kong, China; 4 Royal Derby Hospital, University Hospitals of Derby and Burton NHS Foundation Trust, Derby, UK; 5 Department of Urology, Kaohsiung Medical University Hospital, No. 100, Shih-Chuan 1St Road, Sanmin Dist., Kaohsiung, 80708 Taiwan; 6 Department of Urology, School of Medicine, College of Medicine, Kaohsiung Medical University, No. 100, Shih-Chuan 1St Road, Sanmin Dist., Kaohsiung, 80708 Taiwan; 7 Graduate Institute of Clinical Medicine, College of Medicine, Kaohsiung Medical University, No. 100, Shih-Chuan 1St Road, Sanmin Dist., Kaohsiung, 80708 Taiwan

**Keywords:** Benign prostatic hyperplasia, Alpha-blockers, Storage symptoms, Add-on medications, Network meta-analysis

## Abstract

**Background:**

Patients with benign prostatic hyperplasia (BPH) receive α-blockers as first-line therapy to treat lower urinary tract symptoms; however, some individuals still experience residual storage symptoms. Antimuscarinics, β3-agonists, and desmopressin are effective add-on medications. Nevertheless, there is currently no evidence for the appropriate choice of the first add-on medication. This systematic review aimed to investigate the clinical benefits of antimuscarinics, β3-agonists, and desmopressin, in addition to α-blockers, for persistent storage symptoms in BPH patients.

**Methods:**

A comprehensive literature search of randomized controlled trials (RCTs) comparing the efficacy of different add-on medications in BPH patients with persistent storage symptoms despite α-blocker treatment was conducted. Clinical outcomes included the International Prostate Symptom Score (IPSS), IPSS storage subscore, nocturia, micturition, and urgency. A network meta-analysis was performed to estimate the effect size. Surface under cumulative ranking curves (SUCRAs) were used to rank the included treatments for each outcome.

**Results:**

A total of 15 RCTs were identified. Add-on imidafenacin and mirabegron resulted in significant improvement in all outcomes assessed. Other add-on medications such as desmopressin, tolterodine, solifenacin, fesoterodine, and propiverine showed positive benefits for most, but not all, outcomes. Based on the SUCRA rankings, add-on desmopressin was the best-ranked treatment for IPSS and nocturia, and add-on imidafenacin was the best for the IPSS storage subscore and micturition.

**Conclusions:**

BPH patients presenting with persistent storage symptoms despite α-blocker administration are recommended to include additional treatment. Desmopressin and imidafenacin may be considered high-priority add-on treatments because of their superior efficacy compared with other medications.

## Introduction

Benign prostatic hyperplasia (BPH) is a common condition in the elderly male population, occurring in nearly 70% of men aged > 60 years, and increasing with age [1]. BPH can cause lower urinary tract symptoms (LUTS/BPH) by obstructing the bladder neck, which may be bothersome and have a detrimental impact on the quality of life (QoL). LUTS/BPH has been found to affect 50%-75% of men aged > 50 years, increasing to 80% of men aged > 70 years [2]. For men with moderate-to-severe or bothersome LUTS/BPH, α-blockers are prescribed as first-line pharmacological agents that target the prostate and bladder outlets. Nonetheless, some men with LUTS/BPH fail to respond to α-blockers, particularly those with storage symptoms [3–5].

According to the 2022 International Continence Society (ICS) committee, overactive bladder (OAB) is a complex of storage symptoms defined as urinary urgency, with or without urgency incontinence, usually accompanied by frequency and nocturia [6]. The coexistence of OAB and BPH (OAB/BPH) has been widely identified, and storage symptoms are more bothersome than voiding symptoms [7]. Although α-blockers are administered as the initial treatment for BPH patients with moderate-to-severe LUTS, a subset of patients still experience persistent OAB symptoms of varying degrees of severity, which may be caused by urodynamic detrusor overactivity (DO) or bladder outlet obstruction (BOO) secondary to BPH [8]. The efficacy of different classes of medication added to α-blockers for OAB/BPH has been demonstrated in previous studies. Antimuscarinics were suggested to be added if patients with moderate-to-severe BPH still have residual storage symptoms suggestive of OAB after α-blocker administration, based on the 2018 European Association of Urology Guidelines [9]. β3-agonists such as mirabegron were found to be effective as add-on treatments for OAB symptoms caused by BPH following α-blocker treatment [10–12]. Desmopressin, an antidiuretic agent, added to α-blockers was confirmed as an active therapy in reducing the International Prostate Symptom Score (IPSS) and nocturia episodes in patients not satisfied with α-blocker monotherapy for persistent nocturia [13, 14].

To the best of our knowledge, several medications in different classes have been added to α-blockers for BPH patients with residual OAB symptoms, with variable efficacy and safety outcomes. However, the suitable choice of a second-line add-on agent is currently uncertain. Therefore, we conducted a systematic review and network meta-analysis to investigate the clinical benefits of add-on antimuscarinics, β3-agonists, and desmopressin in patients with BPH and residual OAB symptoms after α-blocker administration.

## Materials and methods

### Search strategy

An electronic search of the MEDLINE and EMBASE databases from inception to 2021 was conducted to identify eligible studies. The search strategy involved the following keywords (MeSH terms and free text words): “benign prostatic hyperplasia,” “overactive bladder,” “α-blockers,” “add-on therapy,” “randomized controlled trial,” and “clinical trial.” Only full-text articles published in English were included. Ongoing trials were identified by searching the Cochrane Controlled Trials Register. The reference lists of the included studies were examined to identify additional studies.

### Study selection

Trials were eligible for inclusion if they were parallel-design randomized controlled trials (RCTs) or crossover studies, included patients diagnosed with BPH receiving α-blockers as initial treatment for at least 4 weeks, and compared any of the following drugs added to α-blockers: desmopressin, imidafenacin, tolterodine, mirabegron, solifenacin, fesoterodine, and propiverine. The outcomes of this study were the IPSS, IPSS storage subscore, nocturia, micturition, and urgency. Trials that included one or more of these outcomes were considered eligible for inclusion. Duplicates were initially removed using reference management software, and two authors independently assessed the eligibility of the remaining studies by sequentially reviewing the titles, abstracts, and full articles.

### Data extraction and quality assessment

Two investigators independently extracted the data using a standardized form. The following data were extracted: study information (title, authors, country, publication time, patient number, and treatment duration), patient characteristics (age, race, bladder diary information, prostate volume, prostate specific antigen, post-void residual volume [PVR], and maximum urinary flow [Q_max_]), intervention, control, and outcomes (estimated effects, standard deviation, standard error, *P*-value, and/or confidence interval [CI]). Quality assessment was performed using the risk of bias assessment tool from the Cochrane Handbook for Systematic Reviews of Interventions [15]. Any discrepancy was resolved by discussion between the two reviewers or by a third reviewer.

## Results

### Literature search

**Fig. 1:**
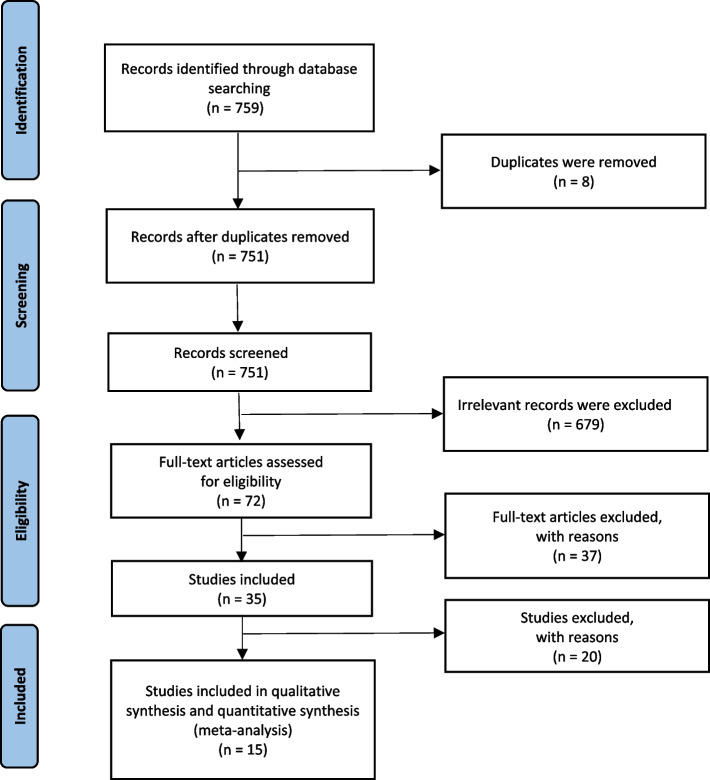
PRISMA flow diagram

A total of 759 studies were identified in the comprehensive literature search. Of these, 8 duplicates were excluded. After the titles and abstracts of 751 studies were reviewed, 679 were removed because of irrelevance, resulting in 72 studies for a full-text review. Finally, 15 studies met our review inclusion criteria and remained for qualitative synthesis and quantitative meta-analysis, including 4875 patients receiving seven different drug therapies. The Preferred Reporting Items for Systematic Reviews and Meta-Analyses (PRISMA) flow diagram is presented in Fig. 1.

### Study characteristics and quality evaluation

The 15 included studies were RCTs lasting 6–12 weeks. Their detailed clinical characteristics are presented in Table 1. All patients in the 15 RCTs were treated with α-blockers before randomization and throughout the trials. For most parallel two-arm RCTs, the effects of drugs of different classes plus α-blockers and α-blockers alone were compared, while the other three-arm RCTs (three studies) tested different doses of the same add-on drug. The add-on treatments identified were desmopressin 0.2 mg (desmopressin + α-blockers), tolterodine 4 mg (tolterodine + α-blockers), mirabegron 50 mg (mirabegron + α-blockers), solifenacin 5 and 10 mg (solifenacin + α-blockers), fesoterodine 4 mg (fesoterodine + α-blockers), propiverine 10 and 20 mg (propiverine + α-blockers), and imidafenacin 0.1 and 0.2 mg (imidafenacin + α-blockers). Kaplan et al. [11] administered dose titrations of mirabegron (titrated from 25 to 50 mg over the last 8 weeks). Furthermore, patients randomized into the fesoterodine arm in the study by Kaplan et al. [11] commenced fesoterodine with optional dose escalation (from 4 to 8 mg) at week 4 and reduction to 4 mg at week 8. All eligible trials involved males with a mean age of 66.79.

**Table 1:** Characteristics of the included studies

**Study**	**Year**	**Study design**	**Initial treatment duration**	**Treatment arm**	**Patient number**	**Add-on intervention duration**
Alquraishi et al.	2020	2-arm RCT	10 wk	*1. α*-blocker *2. α*-blocker + Desmopressin	22 29	4 wk
Chapple et al.	2009	2-arm RCT	4 wk	*1. α*-blocker *2. α*-blocker + Tolterodine	323 329	12 wk
Ichihara et al.	2015	2-arm RCT	8 wk	*1. α*-blocker *2. α*-blocker + Mirabegron	38 38	8 wk
Kakizaki et al.	2019	2-arm RCT	4 wk	*1. α*-blocker *2. α*-blocker + Mirabegron	283 282	12 wk
Kaplan et al.	2020	2-arm RCT	4 wk	*1. α*-blocker *2. α*-blocker + Mirabegron	339 337	12 wk
Kaplan et al.	2009	2-arm RCT	4 wk	*1. α*-blocker *2. α*-blocker + Solifenacin	195 202	12 wk
Kaplan et al.	2012	2-arm RCT	6 wk	*1. α*-blocker *2. α*-blocker + Fesoterodine	472 471	12 wk
Kim et al.	2017	2-arm RCT	8 wk	*1. α*-blocker *2. α*-blocker + Desmopressin	39 47	8 wk
Konstantinidis et al.	2013	2-arm RCT	1 wk	*1. α*-blocker *2. α*-blocker + Fesoterodine	23 24	4 wk
Kwon et al.	2020	2-arm RCT	8 wk	*1. α*-blocker *2. α*-blocker + Mirabegron	19 39	8 wk
Nishizawa et al.	2011	3-arm RCT	8 wk	*1. α*-blocker *2. α*-blocker + Propiverine (10 mg) 3. *α*-blocker + Propiverine (20 mg)	60 60 62	12 wk
Takeda et al.	2013	2-arm RCT	8 wk	*1. α*-blocker *2. α*-blocker + Imidafenacin	154 154	12 wk
Yamaguchi et al.	2011	3-arm RCT	6 wk	*1. α*-blocker *2. α*-blocker + Solifenacin (2.5 mg) 3. *α*-blocker + Solifenacin (5 mg)	212 210 203	12 wk
Yang et al.	2007	2-arm RCT	1 wk	*1. α*-blocker *2. α*-blocker + Tolterodine	36 33	6 wk
Yokoyama et al.	2015	3-arm RCT	4 wk	*1. α*-blocker *2. α*-blocker + Imidafencin (0.2 mg) 3. *α*-blocker + Imidafencin (0.1 mg)	46 43 41	8 wk

### Network meta-analysis

**Fig. 2:**
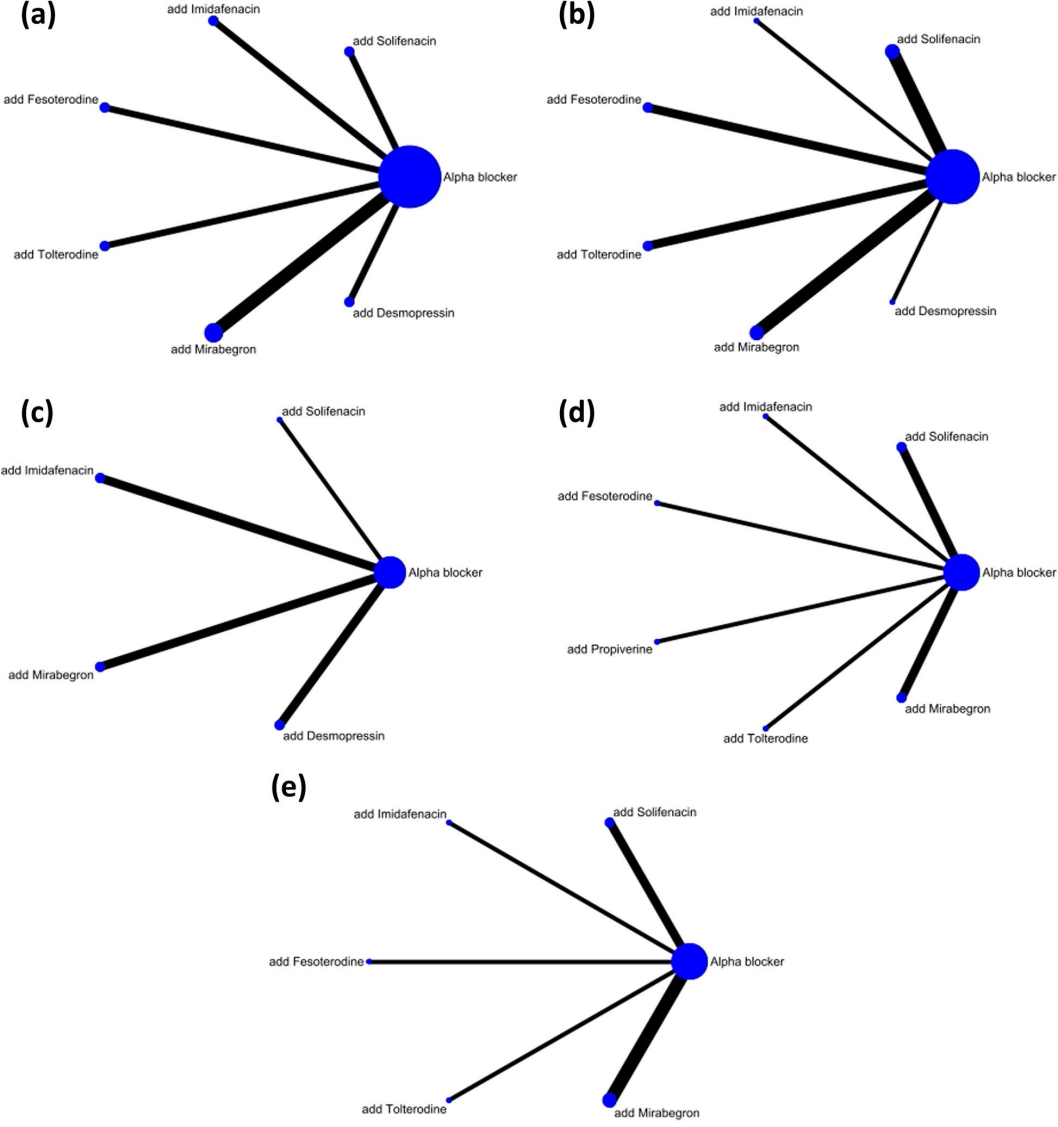
Network constructions for comparison in IPSS total score, IPSS storage subscore, nocturia, micturition, and urgency (**a**) IPSS total score (**b**) IPSS storage subscore (**c**) Nocturia (**d**) Micturition (**e**) Urgency

**Fig. 3:**
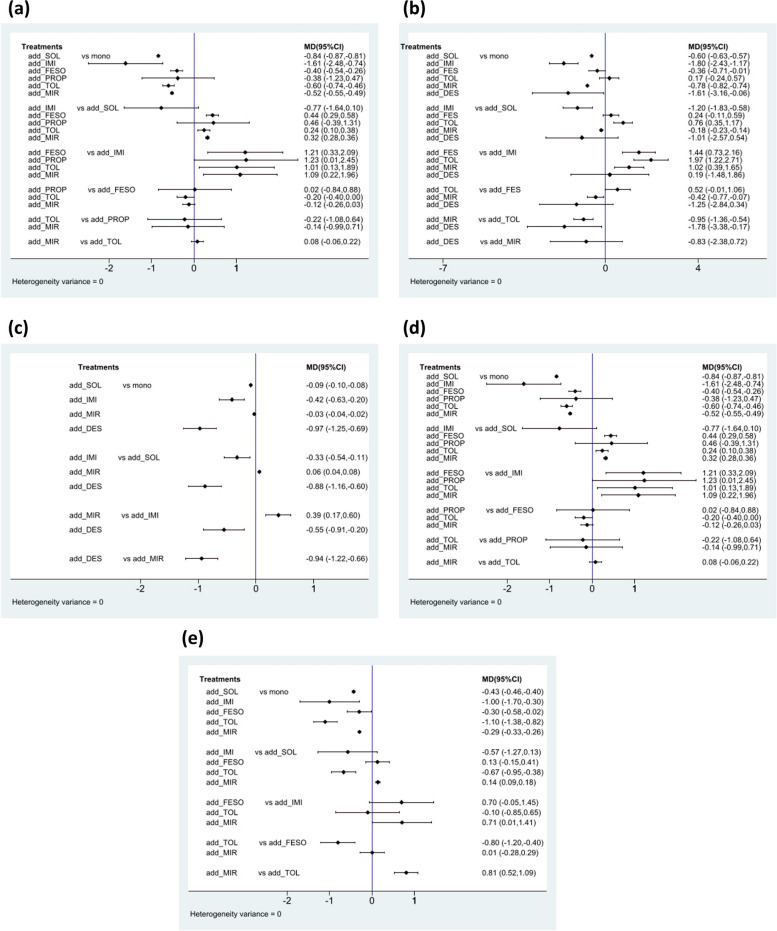
Summary of effect size for pairwise comparison (**a**) IPSS total score (**b**) IPSS storage subscore (**c**) Nocturia (**d**) Micturition (**e**) Urgency

**Fig. 4:**
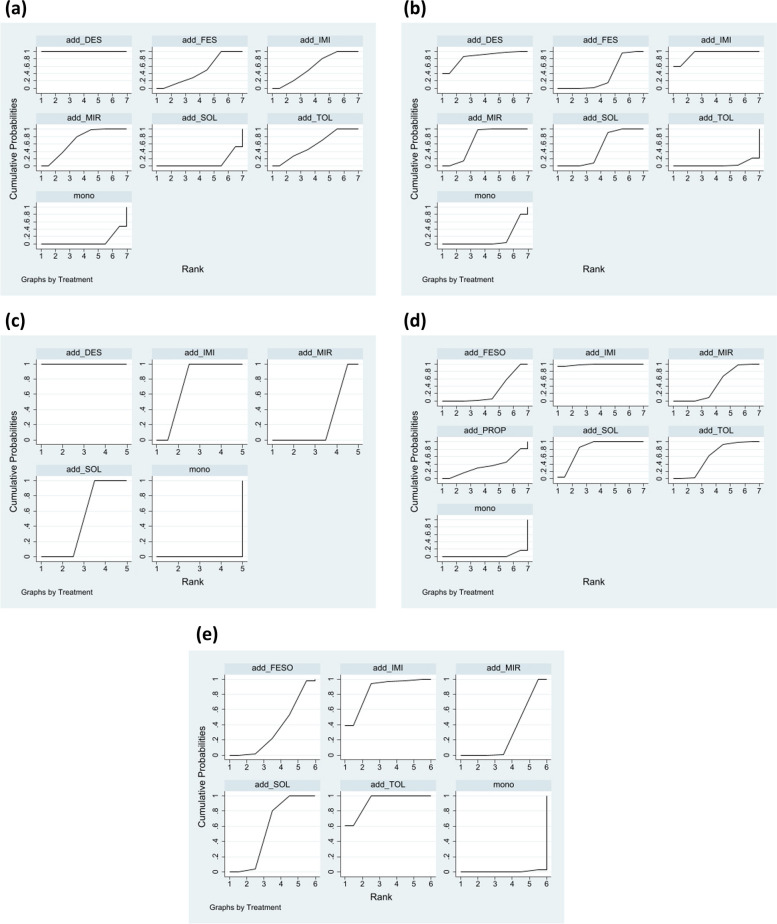
Cumulative ranking probability for different add-on medications (**a**) IPSS total score (**b**) IPSS storage subscore (**c**) Nocturia (**d**) Micturition (**e**) Urgency

A network meta-analysis was performed to assess indirect treatment comparisons. The network constructions for the different outcomes of the IPSS, the IPSS storage subscore, nocturia, micturition, and urgency are shown in Fig. 2. For all clinically assessed outcomes, eight interventions were included in the network analysis, such as α-blockers alone, antimuscarinics + α-blockers (tolterodine + α-blockers, solifenacin + α-blockers, fesoterodine + α-blockers, propiverine + α-blockers, and imidafenacin + α-blockers), beta-3 agonists (mirabegron + α-blockers), desmopressin + α-blockers. Pairwise comparisons of the treatment effect, surface under cumulative ranking curves (SUCRAs), and probability of being the best (Prbest) treatment are shown in Figs. 3 and 4, respectively.

### IPSS score

The results of the IPSS analysis based on the 15 studies are presented in Fig. 2a. Propiverine was not included in this analysis in the original study. Compared to treatment with α-blockers alone, add-on treatment with desmopressin, mirabegron, imidafenacin, tolterodine, and fesoterodine was effective in reducing the total IPSS (Fig. 3a). However, adding solifenacin to α-blockers showed no significant improvement (mean difference: 0.00 [95% CI: -0.06, 0.06]; Fig. 3a). According to the SUCRA results and Prbest score, desmopressin added to α-blockers was the highest-ranked treatment for the total IPSS score (SUCRA = 100%; Prbest = 100%; Fig. 4a), followed by mirabegron (SUCRA = 69%), imidafenacin (SUCRA = 58.3%), tolterodine (SUCRA = 57.1%), and fesoterodine (SUCRA = 48.9%).

### IPSS storage subscore

Further analysis of the IPSS storage subscore was conducted based on seven studies (Fig. 2b). Among the six add-on treatments, imidafenacin, desmopressin, mirabegron, solifenacin, and fesoterodine, with α-blockers, effectively reduced the IPSS storage subscore compared to α-blockers alone (Fig. 3b). No significant difference was found between adding tolterodine to α-blockers and α-blocker monotherapy (mean difference: 0.17 [95% CI: -0.24, 0.57]; Fig. 3b). According to the SUCRA results and Prbest score, imidafenacin added to α-blockers best reduced the IPSS storage subscore (SUCRA = 93.2%; Prbest = 59.3%; Fig. 4b), followed by desmopressin (SUCRA = 84.6%), mirabegron (SUCRA = 68.8%), solifenacin (SUCRA = 49.8%), fesoterodine (SUCRA = 35.6%), α-blockers alone (SUCRA = 13.7%), and tolterodine (SUCRA = 4.3%).

### Nocturia

The nocturia analysis was based on six studies, and the network construction is presented in Fig. 2c. In the original study, tolterodine, mirabegron, fesoterodine, and propiverine were not included in this analysis. Compared to treatment with α-blockers alone, add-on treatment with desmopressin, imidafenacin, solifenacin, and mirabegron was effective in reducing nocturia episodes (Fig. 3c). According to the SUCRA results and Prbest scores, desmopressin may be considered the most successful second-line regimen for nocturia (SUCRA = 100%; Prbest = 99.9%; Fig. 4c), followed by imidafenacin (SUCRA = 74.9%), solifenacin (SUCRA = 50%), and mirabegron (SUCRA = 25%).

### Micturition

The micturition frequency analysis was based on seven studies, and the network construction is shown in Fig. 2d. Desmopressin was not included in this analysis in the original study. Compared to treatment with α-blockers alone, add-on treatment with imidafenacin, solifenacin, tolterodine, mirabegron, and fesoterodine was effective in reducing micturition. However, adding propiverine to α-blockers showed no significant improvement (mean difference: -0.38 [95% CI: -1.23, 0.47]; Fig. 3d). According to the SUCRA results and Prbest score, imidafenacin added to α-blockers was the highest-ranked treatment for micturition (SUCRA = 98.3%; Prbest = 94.4%; Fig. 4d), followed by solifenacin (SUCRA = 81.4%), tolterodine (SUCRA = 59.2%), mirabegron (SUCRA = 45.5%), propiverine (SUCRA = 35.3%), and fesoterodine (SUCRA = 27.3%).

### Urgency

The urgency analysis was based on eight studies, and the network construction is shown in Fig. 2e. In the original study, desmopressin and propiverine were not included in this analysis. All add-on treatments with tolterodine, imidafenacin, solifenacin, fesoterodine, and mirabegron were more effective than α-blocker monotherapy in reducing urgency episodes (Fig. 3e). According to the SUCRA results and Prbest score, the probability of tolterodine added to α-blockers was associated with the highest urgency ranking (SUCRA = 92.2%; Prbest = 60.9%; Fig. 4e), followed by imidafenacin (SUCRA = 85.6%), solifenacin (SUCRA = 56.8%), fesoterodine (SUCRA = 34.9%), and mirabegron (SUCRA = 30%).

## Discussion

Although α-blockers remain the first-line treatment for males with BPH, a subset of patients still exhibit residual OAB symptoms, including urinary urgency, urge incontinence, frequency, and nocturia. OAB symptoms caused by consistent DO may be a possible reason for treatment failure as DO is poorly associated with BOO affected by α-blockers. Thus, there is increasing concern regarding add-on treatments of OAB symptoms in patients with BPH. The benefits and side effects of these medications and their combinations according to the literature are shown in Table 2. The add-on treatment led to a significant improvement in patients with BPH and concomitant OAB in the total IPSS, IPSS storage subscore, IPSS voiding subscore, mean number of micturitions per day, urgency episodes per day, nocturia episodes per day, total overactive bladder symptom score (OABSS), and mean volume voided. In general, add-on treatments appear to be superior to monotherapy in many respects. Although adding a second medication to α-blockers may be helpful for patients experiencing residual OAB, evidence of the comparative effectiveness of different add-on medications is limited in the absence of published head-to-head trials. To compare multiple add-on treatments, a network meta-analysis was developed using direct comparisons of interventions among trials and indirect comparisons across RCTs [16, 17]. Hence, this systematic review and network meta-analysis has assessed the efficacy of a range of medications for treating OAB in improving clinical outcomes as add-on treatments for patients with BPH and residual OAB symptoms despite α-blocker prescription. We included 15 RCTs of seven add-on medications used in 4875 patients. Our results indicate that add-on treatment appears to be more effective than α-blockers alone in improving the total IPSS score, IPSS storage subscore, mean number of micturitions per day, urgency episodes per day, and nocturia episodes per day.

**Table 2:** Benefits and side effects of medications for storage symptoms

Medications	Benefits	Side effects
Antimuscarinics	standard, widely used pharmacotherapy for OAB and effective in men	dry mouth, constipation, blurred vision, dizziness
β3-agonists	favorable tolerability, improved persistence and adherence, and cost-effective comparing with antimuscarinics over long-term treatment	headache, hypertension, tachycardia, constipation
Desmopressin	reducing frequency, urgency, and nocturia by decreasing urinary output and increasing duration of reaching functional bladder capacity	headache, nausea, dizziness, hyponatremia
**Combinations**		
Antimuscarinics + α-blockers	improve urgency, voiding frequency, nocturia, and IPSS without significantly reducing Qmax than α-blockers alone	more frequent side effects than using each drug separately, such as dry month
β3-agonists + α-blockers	greater improvements in OAB symptom score than monotherapy	hypertension, headache, nasopharyngitis
Desmopressin + α-blockers	effective and well tolerated treatment for refractory nocturia	hyponatremia

Patients with BPH and refractory nocturia usually do not completely respond to α-blockers because the relief of bladder outlet obstruction is not sufficient to overcome nocturia. This may be because of the multifactorial mechanisms of nocturia in aging males [18]. Nocturnal polyuria (NP), which is defined as the voided urine volume during the hours of sleep exceeding 33% of the 24-h output, was found to be the main cause of nocturia. NP is a common condition in patients with nocturia (up to 82.9%) [18] and is more prevalent in the elderly population because nocturnal urine production increases with age. Yoong et al. reported that 85% of male patients with nocturia and LUTS with a poor response to α-blockers had NP [19]. NP should be considered a possible cause of refractory nocturia despite α-blocker treatment.

In our analysis, we found that desmopressin added to α-blockers resulted in the greatest improvement in the total IPSS score and nocturia and ranked second in improving the IPSS storage subscore based on the SUCRA. Desmopressin, an arginine vasopressin synthetic analogue, causes similar inhibitory effects on diuresis. It can significantly decrease nocturnal urine output and the number of nocturia episodes [20], which may subsequently improve storage and voiding symptoms, resulting in a decreased IPSS. A systematic review concluded that oral desmopressin added to α-blockers was more effective in improving the IPSS and nocturnal symptoms than using α-blockers alone, with a 64.3% reduction in the frequency of nocturia in comparison with 44.6% [21]. Shin et al. reported a significant decrease in nocturnal urine volume, nocturia episodes, OABSS, urgency episodes, and the nocturnal bladder capacity index when using desmopressin plus α-blockers [22]. Bae et al. showed that the mean number of nocturnal voids, total IPSS, and IPSS storage subscore significantly improved after desmopressin add-on therapy [14]. In addition, add-on desmopressin could improve the QoL of men with BPH, with higher satisfaction with medication and greater willingness to continue treatment [23]. Regarding the safety assessment, the most concerning adverse event of add-on therapy with desmopressin was hyponatremia. Although most patients who developed hyponatremia were asymptomatic, regular assessment of serum sodium levels after initiating desmopressin add-on therapy is recommended, especially in men of advanced age. Owing to the clinical effectiveness and relative safety of desmopressin, the addition of desmopressin to α-blockers may be a suitable therapy for patients with BPH and residual OAB symptoms, especially nocturia.

Although studies have shown that the addition of antimuscarinics to α-blockers is recommended for persistent OAB symptoms associated with BPH [24], comparisons among antimuscarinics are currently unclear. Based on our results, imidafenacin added to α-blockers resulted in the greatest reduction in the IPSS storage subscore and micturition and was also presented as the second-best choice for improving nocturia and urgency based on the SUCRA. The Good-Night study showed that add-on imidafenacin significantly reduced the frequencies of 24 h and nocturnal micturition and nocturnal urine volume in the nightly imidafenacin group (α1-blocker plus 0.1 mg imidafenacin nightly) [25]. Similarly, the ADDITION study reported that add-on imidafenacin (tamsulosin 0.2 mg/d + imidafenacin 0.1 mg twice per day) significantly improved the frequency of daytime urination, nighttime urination, and urinary urgency; IPSS; and total OABSS. A recent meta-analysis also concluded that imidafenacin added to α-blockers significantly improved OAB symptoms and greatly reduced OABSS compared to α-blocker monotherapy [26]. Imidafenacin, an antimuscarinic agent, has a high affinity for M3 and M1 muscarinic receptor subtypes and a low affinity for M2 receptors [27]. In clinical experiments, imidafenacin also inhibits the contraction of detrusor smooth muscles by blocking both postjunctional M3 receptors and prejunctional M1 receptors in humans [28]. The superior efficacy of add-on imidafenacin over other antimuscarinics in the treatment of OAB symptoms may be explained by its unique pharmacological effects. Imidafenacin has a shorter half-life (2.9 h), relatively greater selectivity, and longer duration of receptor binding in the bladder than in the salivary gland and other organs in rats (6–9 h in the bladder, 1–3 h in the submaxillary gland; no observation in the brain) [29, 30] compared with other antimuscarinic agents. Interestingly, our results also revealed that add-on imidafenacin showed the greatest improvement in nocturia compared with other antimuscarinics. This finding was consistent with previous studies, which speculated that imidafenacin may reduce the number of nighttime voids, increase bladder capacity, and improve sleep disorders [25, 31]. In an animal experiment, Watanabe et al. showed that imidafenacin decreased urine volume by suppressing the C-fibers in the rat bladder [32]. A possible mechanism by which imidafenacin improves nocturia is that it decreases nocturnal urine volume by inhibiting bladder afferent nerves, causing subsequent improvement in nocturia and sleep disturbance [25]. In terms of safety, imidafenacin has fewer adverse events such as dry mouth and constipation than other antimuscarinic agents [33, 34], which could be explained by its higher selectivity for the bladder. Furthermore, Wu et al. reported that imidafenacin was associated with a significantly lower withdrawal rate related to adverse events [34]. There have been concerns that antimuscarinic add-on may theoretically aggravate voiding symptoms by inhibiting detrusor muscle contraction, resulting in reduced Q_max_ improvements, increased PVR, and, in particular, acute urinary retention. However, there were no significant differences in Q_max_ or PVR after the addition of imidafenacin to α-blockers [26, 35]. Collectively, imidafenacin add-on treatment was effective, safe, and well-tolerated for residual OAB symptoms in patients with BPH already receiving α-blockers, with superior efficacy in improving micturition, urgency, and nocturia compared with other antimuscarinic agents.

Our results indicated that add-on mirabegron was effective in treating residual OAB symptoms such as micturition, urgency, and nocturia in patients already receiving α-blockers, ranking second in improving the IPSS score and third in the IPSS storage subscore based on the SUCRA. Previous studies have corroborated our findings. Two RCTs reported that adding mirabegron to tamsulosin significantly improved the total IPSS, IPSS storage subscore, and total OABSS [12, 36]. Kaplan et al. reported that the addition of mirabegron to tamsulosin significantly improved micturition, urgency, total urgency, and frequency scores [11]. A recent meta-analysis showed that add-on mirabegron therapy significantly reduced the mean number of micturition episodes, urgency episodes per day, and total OABSS compared with tamsulosin monotherapy [10]. Mirabegron was also proven urodynamically efficacious and safe for treating men with BPH and OAB. Add-on mirabegron treatment significantly increased the Q_max_ and voided volume [37, 38]. These findings may be attributed to the pharmacological characteristics of mirabegron. Mirabegron, as a β3-agonist, not only promotes relaxation of the detrusor smooth muscle to increase bladder capacity [39] but also shows competitive antagonist activity on the α_1_-adrenoceptors in the urethra, resulting in urethral smooth muscle relaxation [40]. Regarding the safety assessment, the incidence rates of treatment-emergent adverse events (TEAEs) with mirabegron added to α-blockers and α-blockers alone were similar, and TEAEs were mild in severity [10, 41]. Although an increase in PVR was observed with add-on mirabegron treatment in some studies, the change in PVR was not clinically meaningful [41]. Mirabegron appears to be a safe treatment option for patients with predominantly coexisting OAB and BPH after receiving α-blockers. Furthermore, patients receiving mirabegron have significantly higher persistence and adherence rates than those treated with antimuscarinics, with lower occurrence of TEAEs including dry mouth and constipation [42, 43]. Because add-on mirabegron treatment exhibited satisfactory efficacy and safety and was well-tolerated, it could be an alternative choice for treating residual OAB symptoms in patients with BPH who have used α-blockers and are not satisfied with other add-on medications.

## Limitation

To the best of our knowledge, this is the first network meta-analysis to compare the efficacy of different medications as add-on treatments to α-blockers in patients with BPH and concomitant OAB. However, this study had some limitations. First, the results of our study were short-term outcomes, with the duration of the add-on interventions not exceeding 12 weeks. Furthermore, high-quality RCTs are required to determine the long-term efficacy and persistence of these add-on medications. Second, safety outcomes were not included in our study; therefore, the potential risk of adverse events remains. Third, various types of α-blockers were included in our analysis, which may have affected the results owing to the different α1-adrenergic receptor subtype selectivities of α-blockers. However, our primary aim was to examine the additional benefits of add-on medications. Furthermore, not all RCTs included in the present study evaluated the full range of urodynamic parameters, although urodynamic examination may provide important information related to bladder and urethral dysfunction.

## Conclusions

Our network meta-analyses showed that BPH patients, who presented with persistent storage symptoms despite α-blocker administration, are recommended additional treatment. Desmopressin and imidafenacin may be considered high-priority add-on treatments because of their superior efficacy compared with other medications. Further RCTs are needed to evaluate the long-term outcomes.

## Data Availability

The data supporting the findings of this article are available from the corresponding author on reasonable request.

## Abbreviations

BPH: Benign prostatic hyperplasia
LUTS: Lower urinary tract symptoms
RCT: Randomized controlled trial
IPSS: International Prostate Symptom Score
SUCRA: Surface under cumulative ranking curve
QoL: Quality of life
OAB: Overactive bladder
DO: Detrusor overactivity
BOO: Bladder outlet obstruction
PVR: Post-void residual volume
Q_max_: Maximum urinary flow
CI: Confidence interval
ROB: Risk of bias
PRISMA: Preferred Reporting Items for Systematic Reviews and Meta-Analyses
Prbest: Probability of being best
OABSS: Overactive Bladder Symptom Score
MVV: Mean volume voided
NP: Nocturnal polyuria
TEAE: Treatment-emergent adverse event
